# Astrocyte Reactivity Polygenic Risk Score May Predict Cognitive Decline in Alzheimer’s Disease

**DOI:** 10.1142/9789819807024_0035

**Published:** 2025

**Authors:** Jared M Phillips, Julie A Schneider, David A Bennett, Paul K Crane, Shannon L Risacher, Andrew J Saykin, Logan C Dumitrescu, Timothy J Hohman

**Affiliations:** 1 Vanderbilt Memory and Alzheimer’s Center, Vanderbilt University Medical Center, Nashville, TN, USA; 2 Department of Pharmacology, Vanderbilt University School of Medicine, Nashville, TN, USA; 3 Rush Alzheimer’s Disease Center, Rush University Medical Center, Chicago, IL, USA; 4 Department of Medicine, University of Washington, Seattle, WA, USA; 5 Indiana Alzheimer’s Disease Research Center, Indiana University School of Medicine, Indianapolis, IN, USA; 6 Stark Neurosciences Research Institute, Indiana University School of Medicine, Indianapolis, IN, USA; 7 Vanderbilt Genetics Institute, Vanderbilt University Medical Center, Nashville, TN, USA

**Keywords:** Alzheimer’s disease, polygenic risk, astrocyte reactivity, cognition, biomarkers

## Abstract

Alzheimer’s disease (AD) is a polygenic disorder with a prolonged prodromal phase, complicating early diagnosis. Recent research indicates that increased astrocyte reactivity is associated with a higher risk of pathogenic tau accumulation, particularly in amyloid-positive individuals. However, few clinical tools are available to predict which individuals are likely to exhibit elevated astrocyte activation and, consequently, be susceptible to hyperphosphorylated tau-induced neurodegeneration. Polygenic risk scores (PRS) aggregate the effects of multiple genetic loci to provide a single, continuous metric representing an individual’s genetic risk for a specific phenotype. We hypothesized that an astrocyte activation PRS could aid in the early detection of faster clinical decline. Therefore, we constructed an astrocyte activation PRS and assessed its predictive value for cognitive decline and AD biomarkers (i.e., cerebrospinal fluid [CSF] levels of Aβ1–42, total tau, and p-tau181) in a cohort of 791 elderly individuals. The astrocyte activation PRS showed significant main effects on cross-sectional memory (β = −0.07, p = 0.03) and longitudinal executive function (β = −0.01, p = 0.03). Additionally, the PRS interacted with amyloid positivity (p.intx = 0.02), whereby indicating that amyloid burden modifies the association between the PRS and annual rate of language decline. Furthermore, the PRS was negatively associated with CSF Aβ1–42 levels (β = −3.4, p = 0.07) and interacted with amyloid status, such that amyloid burden modifies the association between the PRS and CSF phosphorylated tau levels (p.intx = 0.08). These findings suggest that an astrocyte activation PRS could be a valuable tool for early disease risk prediction, potentially enabling intervention during the interval between pathogenic amyloid and tau accumulation.

## 1. Introduction

Alzheimer’s disease (AD) is a highly polygenic condition characterized by a neuropathological sequence of extracellular amyloid-beta plaques and intracellular neurofibrillary tangles that leads to neurodegeneration and cognitive decline [12]. A distinguishing feature of AD is its prolonged prodromal phase, during which pathology accumulates well before clinical symptoms manifest [2, 14]. This prodromal period spans decades of pathological changes prior to the onset of noticeable cognitive deficits, making early diagnosis of clinical dementia both challenging and crucial in developing precision interventions. Polygenic risk scores (PRS) of AD have displayed some utility in predicting the global genetic risk of developing AD [5] yet demonstrate mixed success clinically [8, 10, 22, 26]. This may be partly due to the case-control genome-wide association study (GWAS) designs used to generate summary statistics that enable PRS calculation, which lack the phenotypic specificity needed to move towards precision interventions.

Astrocyte activation plays a varied and complex role in AD, with numerous detrimental functions that may contribute to disease pathogenesis including induction of tau hyperphosphorylation, impairment of glutamate and ion buffering abilities, and weakening of the neurovascular unit [13, 15, 16, 28]. Recent evidence has emerged that highlights astrocyte activation as an important cellular event linking initial amyloid pathology with subsequent phosphorylated tau accumulation [3]. Most notably, recent findings leveraging *in vivo* measurements of peripheral glial fibrillary acidic protein (GFAP), a strong correlate of astrocyte activation, found that high plasma GFAP expression, representing a greater degree of astrocyte reactivity, relates to higher AD neuropathological burden [3, 29]. This association was most pronounced in amyloid-positive individuals [3]. In acute brain injury, astrocyte reactivity is both beneficial and detrimental, contributing significantly to post-traumatic tissue repair and synaptic remodeling in conditions such as traumatic brain injury and stroke [4] while also facilitating release of pro-inflammatory factors that may exacerbate cognitive decline [19]. As such, the level of chronic astrocyte activation, particularly in the presence of amyloid pathology, may influence an individual’s risk of subsequently developing tau pathology and dementia. Heterogeneity in astrocyte responses to trauma, whether acute or chronic, points to genetic factors that may influence the molecular response of astrocytes to insult [4, 24]. Consequently, investigating the genetic architecture of astrocyte activation in the context of AD may yield insights beneficial in advancing targeted interventions for individuals at risk of developing the detrimental effects of long-term reactive states.

In this study, we sought to accomplish three main aims: 1) to elucidate the genetic architecture of an astrocyte activation phenotype, 2) to build a PRS of astrocyte activation, and 3) to test its ability to predict cognitive decline and associations with AD biomarker levels. Using post-mortem measures of mRNA sequencing from the dorsolateral prefrontal cortex, we calculated an established astrocyte activation transcript signature [33]. Then, we employed this transcript signature as an outcome in GWAS to identify genetic signals associated with the astrocyte activation phenotype. Finally, we built a PRS in an independent dataset to test its associations with cognitive performance in multiple domains and AD biomarker burden.

## 2. Methods

### 2.1. Participants

Participants were sourced from two well-characterized cohort studies of aging, including the Religious Orders Study/Rush Memory and Aging Project (ROS/MAP) and the Alzheimer’s Disease Neuroimaging Initiative (ADNI). Data collection commenced in 1994 for ROS and in 1997 for MAP, resulting in extensive longitudinal clinical-pathologic data on aging and AD risk factors. ROS includes religious clergy members from across the United States, while MAP includes individuals from northeastern Illinois. Initiated in 2003, ADNI encompasses over 1800 individuals between 55 to 90 years old, through four study phases, with the principal objective of validating biomarkers for Alzheimer’s disease clinical trial applications (http://adni.loni.usc.edu/). All participants provided informed consent and the studies were carried out in accordance with Institutional Review Board-approved protocols. The Vanderbilt University Medical Center Institutional Review Board authorized secondary analyses of the data. Data were accessed and harmonized as part of the Alzheimer’s Disease Sequencing Project Phenotype Harmonization Consortium (https://adsp.niagads.org/). Please see Table 1 for an overview of each cohort’s participant demographics.

### 2.2. Cerebrospinal fluid biomarker measures

Lumbar puncture was performed as described in the ADNI procedures manual (http://www.adni-info.org/). CSF measures of β-amyloid(1–42) were obtained using the xMAP platform and CSF measures of total tau and p-tau 181 were obtained using the Elecsys platform. Amyloid positivity was defined as CSF β-amyloid(1–42) concentrations lower than 192 pg/mL as outlined previously [31].

### 2.3. Neuropsychological composites

Harmonized scores representing composite memory, executive function, and language were used in the present analyses and have been previously described in detail [25]. Briefly, this harmonization process involved experts assigning individual test item-level data into memory, executive function, language, visuospatial, or “none of” domains. Investigators ensured identical scoring of anchor items across studies and a confirmatory factor analysis was conducted to choose the best single factor or bi-factor model. Anchor items were items identified as having been administered and scored precisely the same way in two or more cohorts. All items had freely estimated parameters, with anchor items forced to have the same parameters across studies. We used these co-calibrated parameters for anchor and study-specific items to generate cognitive scores that were on the same scale across cohorts.

### 2.4. Genetic data quality control and imputation

For ADNI, genetic data were collected with four arrays (Illumina Human610-Quad, Illumina HumanOmniExpress, Illumina Omni 2.5 M, and Illumina Global Screening Array v2). For ROSMAP, genetic data were collected with three arrays (Global Screening Array-24 v3.0, Affymetrix GeneChip 6.0, Illumina HumanOmniExpress). All genetic data were processed using a standardized quality control and imputation pipeline [7]. First, variants which had a low genotype rate (<95%), low minor allele frequency (MAF<1%) or were outside of Hardy-Weinberg equilibrium (*p*<1×10^−6^) were removed. Participants were excluded if the reported and genotypic sex differed, if there was poor genotyping efficiency (missing>1% of variants), or cryptic relatedness was present (*PIHAT*>0.25). Imputation was performed on the University of Michigan Imputation Server using the TOPMed reference panel (hg38) with SHAPEIT phasing [6, 11, 32]. Following imputation, datasets were filtered to exclude variants with low imputation quality (*R*^*2*^<0.8), duplicated/multi-allelic variants, and MAF<1%. Within the self-identified non-Hispanic White racial group, principal component analysis was conducted and genetic ancestry outliers relative to a 1000 Genomes reference population (eg. Utah residents with Northern and Western European Ancestry [CEU]) were excluded.

### 2.5. Autopsy measures of DLPFC bulk mRNA expression

A standardized protocol for post-mortem biological specimens was used consistently across centers performing autopsies, as previously described [1]. RNA extraction from specific brain regions was conducted using a Qiagen miRNeasy mini kit along with a RNase-free DNase Set for quantification on a Nanodrop. The integrity and purity of the RNA were assessed using an Agilent Bioanalyzer. Samples with a RIN score greater than five were included for bulk next-generation RNA sequencing.

Sequencing was performed in multiple phases. Phase one focused on the dorsolateral prefrontal cortex (dlPFC). Phase two added more dlPFC samples and included samples from the posterior cingulate cortex (PCC) and the head of the caudate nucleus (CN). Phase three included additional participant samples from the dlPFC. Detailed information on RNA processing and sequencing is available on Synapse (syn3388564). In summary, phase one employed poly-A selection, strand-specific dUTP library preparation, and Illumina HiSeq with 101 bp paired-end reads, achieving a coverage of 150 million reads for the first 12 reference samples. These deeply sequenced reference samples included 2 males and 2 females from non-impaired, mild cognitive impairment, and Alzheimer’s disease cases. The remaining samples were sequenced with a coverage of 50 million reads. Phase two used the KAPA Stranded RNA-Seq Kit with RiboErase (kapabiosystems) for ribosomal depletion and fragmentation. Sequencing for this phase was performed on an Illumina NovaSeq6000 with 2 × 100 bp cycles, targeting 30 million reads per sample. In phase three, RNA was extracted with a Chemagic RNA tissue kit (Perkin Elmer, CMG-1212) using a Chemagic 360 instrument, and ribosomal RNA was depleted using RiboGold (Illumina, 20,020,599). Sequencing for phase three was carried out on an Illumina NovaSeq6000 with 40–50 million 2 × 150 bp paired-end reads.

Data processing and QC of RNA sequencing runs was performed by the Vanderbilt Memory and Alzheimer’s Center Computational Neurogenomics Team using an automated pipeline and is described in detail elsewhere [30]. Samples whose last visit was >5 years before death or who had non-AD dementia were excluded.

### 2.6. Statistical analyses

**Figure 1.**
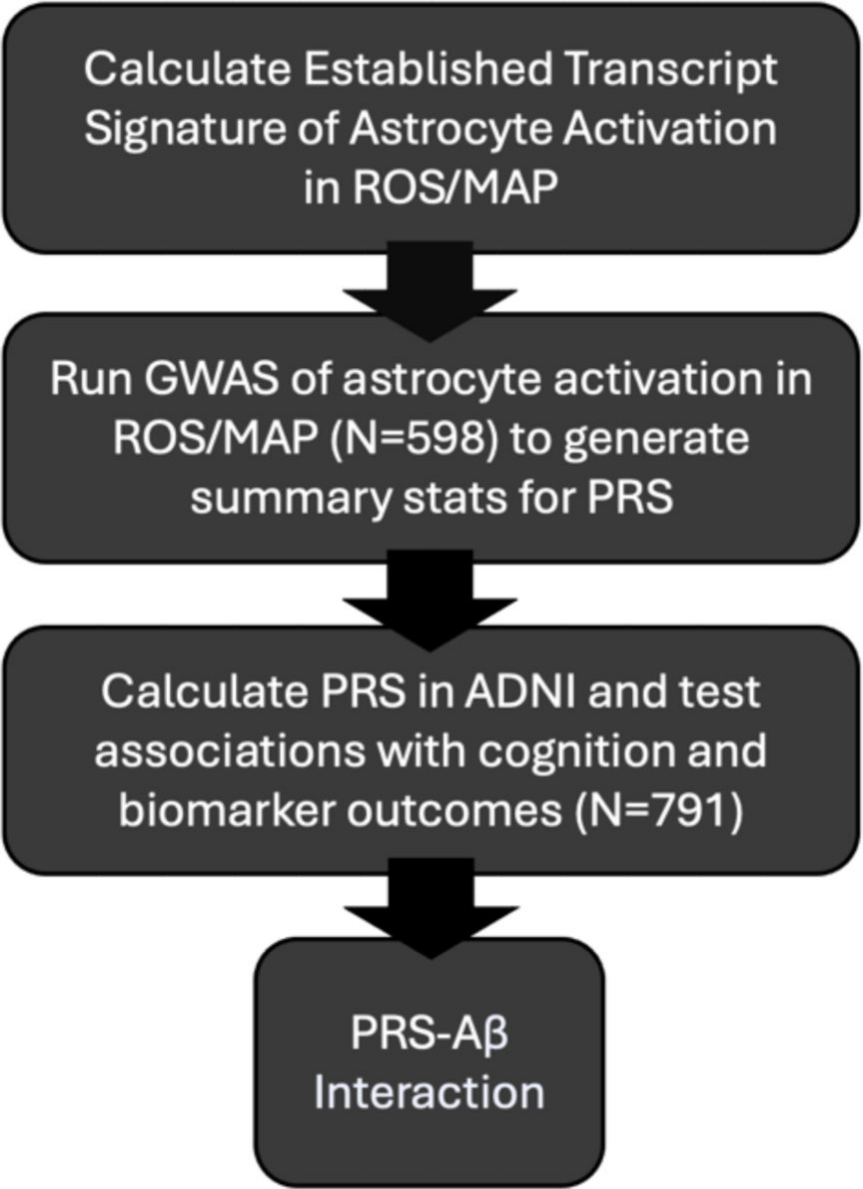
Workflow outlining analytical activities.

See Figure 1 for an overview of analytical activities.

#### 2.6.1. Astrocyte reactivity z-score calculation

Methods for generating an astrocyte reactivity z-score were derived from procedures reported by Wu et al [33]. Briefly, single-nucleus RNA sequencing measures from the dorsolateral prefrontal cortices of 24 participants, representing 162,562 individual nuclei, were clustered into transcriptionally similar clusters using a k-nearest neighbor graph. Further dimensionality reduction occurred through t-SNE and expression of canonical genes, including *AQP4* for astrocytes, was used to identify cell type clusters. This analysis was then repeated within the astrocyte cluster, resulting in ten astrocyte sub-clusters. Next, the expression of genes characteristic of reactive astrocytes as reported in Zamanian et al [34]., including *GFAP*, *CD44*, *OSMR*, and *CHI3L1*, was surveyed, resulting in the identification of three sub-clusters that displayed high expression of all four genes. Differential gene expression was assessed using Seurat to obtain marker genes for these activated astrocyte clusters. Genes were required to be expressed in at least 10% of nuclei in the given cluster and at least log(0.25)-fold difference between the clusters.

Genes that were significantly over-expressed in reactive astrocytes compared to both other astrocyte clusters and all other cells were preserved in the marker gene-set (n=25). Next, we obtained normalized bulk mRNA sequence counts from the ROS/MAP dorsolateral prefrontal cortex dataset, which did not overlap with the snRNA sequencing dataset used to identify reactive astrocyte markers. Four genes were unavailable due to quality control filtering, resulting in 21 genes in the final gene set. Participants with values for all 21 genes were included, leading to a sample size of 843 individuals. Finally, a summary z-score representing higher or lower-than-average reactive astrocyte gene expression was calculated to leverage as an outcome in downstream analyses.

#### 2.6.2. Genome-wide association study of astrocyte reactivity

Following generation of the astrocyte reactivity z-score, we conducted a GWAS to assess the effect of genetic variants on astrocyte reactivity. GWAS were performed with PLINK linear association models (v1.90b5.2, https://www.cog-genomics.org/plink/1.9). 646 participants in ROS/MAP had both genetic data and an astrocyte reactivity z-score. We excluded a random sample of 48 participants from GWAS to later assess the correlation of the astrocyte reactivity z-score and PRS in these individuals, resulting in a final sample size of 598 participants in GWAS. GWAS covariates included RNA-sequencing batch, RNA sequencing sample collection phase, age at death, sex, and the first five principal components of genetic ancestry.

#### 2.6.3. Polygenic risk score generation

No participants in ADNI were included in the astrocyte reactivity GWAS. First, GWAS variants were compared to the ADNI genetic data. Any ambiguous, palindromic variants were filtered out. Then overlapping variants between the GWAS and the ADNI genetic data were retained and subsequently compared for variants on opposite strands between the GWAS and the genetic data, and strand differences were resolved. Then, linkage disequilibrium (LD) clumping was performed with PLINK in the ADNI genetic data (r^2^=0.5, window=250kb), to choose the variant with the most significant phenotypic association within each genetically-linked genomic region. Each PRS was built with three different P-value thresholds: P=0.01, P=0.001, and P=0.00001, wherein variants were included in the PRS only if their phenotypic association was less than the given threshold. The LD-clumped genetic data were then leveraged to calculate each PRS with PLINK’s profile function which calculates scores as follows: Weights were retrieved from the variant associations with AD or with resilience from the respective GWAS. For each variant the given weight was multiplied by 0, 1, or 2, based on how many risk alleles an individual possessed. The summation of this process results in a summary score for an individual.

Since *APOE* polymorphism is a robust risk factor for AD, PRS were calculated with and without the *APOE* region, defined by a 1Mb region up and downstream of the *APOE* gene.

#### 2.6.4. Baseline and longitudinal linear association models

We performed a series of linear fixed and linear mixed effects models in R (v. 4.1.2) for each PRS. Fixed effects in our models included baseline age, sex, and the given PRS. Longitudinal linear mixed effects models included a PRS-by-interval term, where interval was determined by the difference between a participant’s age at each cognitive visit and their baseline age. Additionally, linear mixed effects models allowed slope and intercept to vary for each participant. In addition, we performed identical sets of models with the addition of a PRS-by-amyloid term in linear models and a PRS-by-amyloid-by-interval term for linear mixed effects models, with amyloid measured by the CSF Aβ1–42 assay outlined above. Biomarker-based outcomes of our models were cross-sectional CSF Aβ1–42, CSF total tau, CSF p-tau 181. Cognition-based outcomes of our models were baseline memory, executive function, and language, or longitudinal decline in memory, executive function, and language, using linear and linear mixed effects models, respectively. We re-ran significant or near-significant interaction models as amyloid-stratified models to obtain main effect statistics for amyloid positive (N=527) and amyloid negative (N=257) individuals. We also conducted sensitivity analyses using data-driven cutpoints determined by Gaussian mixture modeling (GMM) to reevaluate amyloid positivity within our sample (amyloid positivity defined as CSF β-amyloid(1–42) concentrations lower than 195 pg/mL; amyloid positive N = 520, amyloid negative N = 264).

## 3. Results

The 21 genes included in the astrocyte activation gene module were positively correlated with one another, with the exceptions of *ARGHEF3* and *ZFYVE28* (Supplemental Figure 1). We subsequently ran GWAS to generate summary statistics to be leveraged in the PRS calculation. GWAS results highlighted loci on chromosomes 2, 6, 7, and 11 with an acceptable genomic inflation factor of 1.0 (Supplemental Figure 2). To evaluate the correlation of each PRS with the astrocyte reactivity Z-score, we built the PRS with a variety of p-value cutoffs in a subset of 48 random participants in ROS/MAP who possessed astrocyte reactivity Z-scores but were excluded from GWAS. The correlation was by far the strongest in the PRS with p-value cutoff < 0.01 (0.98; see Supplemental Figure 3). As such, subsequent analyses focused only on PRS with this p-value cutoff. The correlation between the PRS and astrocyte activation z-score did not differ when excluding the *APOE* region, and no strong loci were observed in the *APOE* region at the GWAS level (Supplemental Figure 2 and Supplemental Figure 3). Consequently, we leveraged PRS which included the *APOE* region in proximate analyses.

**Figure 2.**
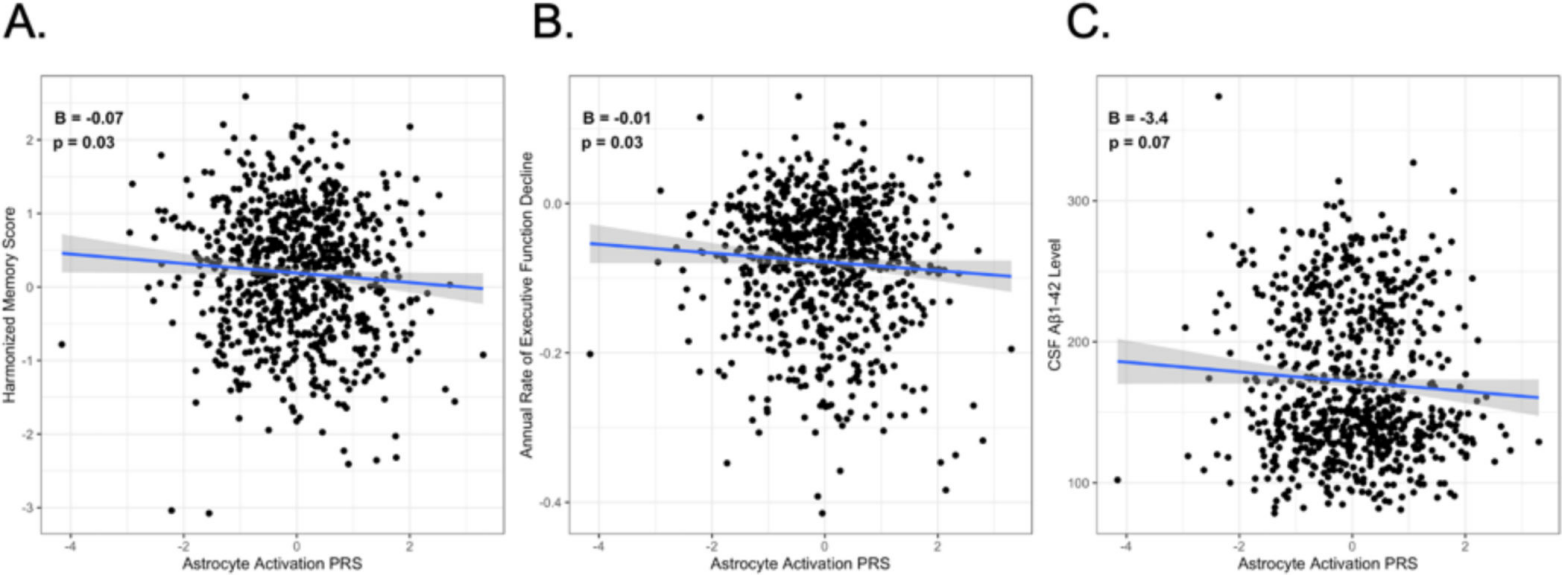
PRS associations with cross-sectional memory, annual rate of executive function decline, and CSF Aβ1–42 level.

We then built the PRS in an independent dataset and evaluated its associations with cross-sectional and longitudinal cognition as well as cross-sectional AD biomarker levels, including CSF Aβ1–42, total tau, and phosphorylated tau. All main effects on cognition and biomarker outcomes are presented in Table 2 and/or Figure 2. The astrocyte activation PRS had significant effects on both cross-sectional memory (Figure 2A) and longitudinal executive function (Figure 2B), such that a higher PRS was associated with worse cross-sectional memory performance and a faster rate of executive function decline. In addition, the PRS was negatively associated with the CSF Aβ1–42 level (Figure 2C), although this result was just below the significance threshold.

**Figure 3.**
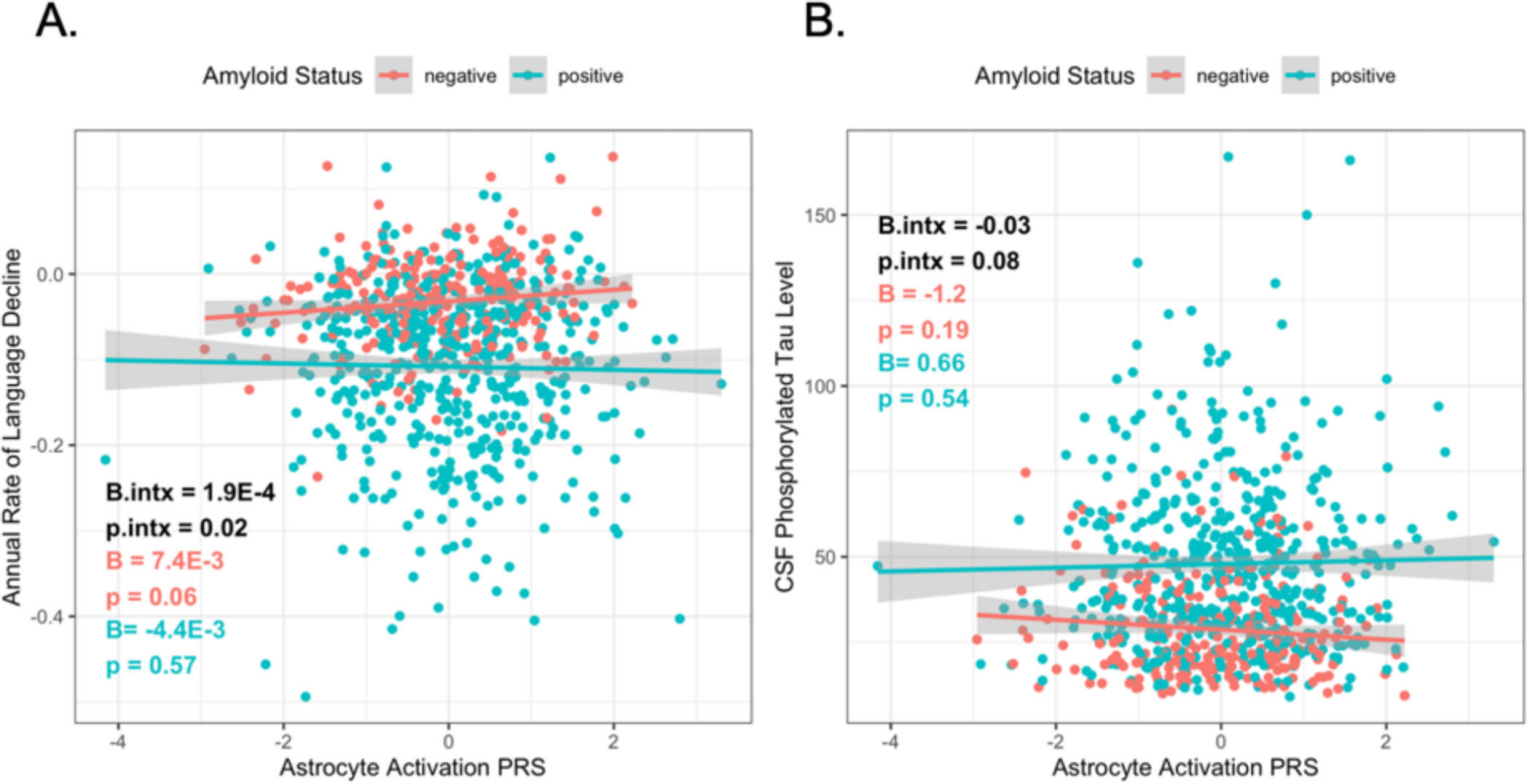
PRS-Aβ42 interactions on annual rate of language decline and CSF phosphorylated tau. Interaction model statistical results are shown in black while amyloid-stratified main effect statistics are shown in colors corresponding to each stratification on the plot.

Next, we performed a series of interaction models to determine if amyloid status modified the effect of the PRS on each outcome (Table 3 and Figure 3). Effects of the PRS on annual rate of language decline differed across amyloid status, and amyloid-negative individuals largely drove the significant interaction (Figure 3A). Effects of the PRS on CSF phosphorylated tau level also differed across amyloid status, with the near-significant interaction being driven by deviations between amyloid-negative and amyloid-positive individuals with higher PRS (Figure 3B). Results were consistent across both the predefined amyloid positivity threshold and the threshold generated through GMM (Supplemental Figure 4). Together, these results suggest a differential effect of the PRS when stratified by amyloid status.

## 4. Discussion

The findings from our study underscore the potential of an astrocyte activation polygenic risk score (PRS) in the preclinical detection and risk stratification of Alzheimer’s disease (AD).

Together, our results highlight several critical points that add to the growing body of literature on the role of astrocytes in AD pathology and suggest practical applications for astrocyte activation PRS in clinical settings.

### 4.1. Genetic architecture of astrocyte activation

We leveraged an established transcript signature of astrocyte activation to serve as a single, continuous outcome in GWAS. Interestingly, the top locus, rs17416058, located on chromosome 11, is an expression quantitative trait locus in brain for *ARNTL* (alias: *BMAL1*), a circadian clock gene (Sources: Braineac and BrainSeq databases). Astrocyte-specific deletion of *BMAL1* has been shown to induce astrocyte activation, indicating a crucial role of circadian rhythm in regulating astrocytic gene expression [18]. Furthermore, astrocytes deficient in *BMAL1* display an enhanced response to amyloid-beta pathology, signaling disease-relevant changes in the face of altered gene expression [23]. Carriage of the minor allele is associated with decreased expression of *BMAL1* in the BrainSeq hippocampus dataset and a higher astrocyte activation transcript signature (β = 0.25, p = 1.3E-7), which is in line with the observed direction of effect in the aforementioned biological literature. As such, *BMAL1* may represent an important genomic locus influencing an individual’s degree of astrocyte reactivity, though this finding requires validation in a well-powered dataset.

### 4.2. Predictive utility of astrocyte activation PRS

The constructed astrocyte activation PRS demonstrated predictive value for cognitive decline, providing a potential genetic tool to anticipate AD progression. The significant associations between higher PRS and both cross-sectional memory (β = −0.07, p = 0.03; Figure 2A) and longitudinal executive function decline (β = −0.01, p = 0.03; Figure 2B) suggest that individuals with a higher genetic predisposition for astrocyte activation exhibit worse cognitive performance cross-sectionally and over time. These findings align with previous research indicating that astrocyte reactivity exacerbates neurodegeneration and cognitive impairment [9, 17, 27, 29]. Furthermore, the negative associations between the astrocyte activation PRS and CSF amyloid-beta 1–42 levels (β = −3.4, p = 0.07; Figure 2C) provide additional insights into the biological underpinnings of AD. Although the result was marginally below the significance threshold, it suggests that higher genetic risk for astrocyte activation is associated with lower CSF amyloid-beta 1–42 levels, potentially reflecting greater amyloid plaque burden in the brain. This association aligns with the hypothesis that astrocyte activation is linked to amyloid pathology and subsequent neurodegenerative processes [3].

### 4.3. Interaction with amyloid positivity

The interaction between the astrocyte activation PRS and amyloid positivity highlights a nuanced understanding of AD pathology. In the case of annual rate of language decline, the significant interaction appears to largely be driven by the effect in amyloid-negative individuals, such that higher PRS relates to a slower rate of language decline (Figure 3A). We observed a smaller effect in amyloid-positive individuals, though both stratifications aligned with the anticipated directions of effect. In the case of CSF phosphorylated tau levels, a stronger effect was also observed in amyloid-negative individuals (Figure 3B). However, the difference in the directions of effect between amyloid-negative and amyloid-positive individuals drives the near-significant interaction. This suggests that the astrocyte activation PRS may identify individuals who are more susceptible to tau pathology in the presence of amyloid accumulation and a potential protective effect of astrocyte activation in the absence of amyloid pathology. It is plausible that increased astrocyte reactivity in the absence of amyloid pathology may lead to decreased neurodegeneration and subsequent cognitive decline, as reactive astrocytes are known to excrete various growth factors that maintain neuronal and synaptic integrity [20]. However, further interrogating this effect would require more precise transcriptional and morphological profiling of reactive astrocytes in the presence and absence of amyloid pathology, an area ripe for future investigation.

### 4.4. Clinical implications and future directions

The astrocyte activation PRS holds promise as a clinical tool for early AD risk stratification and intervention. By identifying individuals at higher genetic risk for astrocyte activation, clinicians can better predict the trajectory of cognitive decline and tailor preventive strategies accordingly. Furthermore, the PRS can aid in the selection of candidates for clinical trials targeting astrocyte-mediated pathways, thereby enhancing the precision and efficacy of therapeutic interventions. Future research should focus on refining the astrocyte activation PRS by genetically surveying the astrocyte activation transcript signature in larger, harmonized datasets to increase statistical power at the GWAS level. Validation of its predictive power in large, diverse cohorts would also be greatly beneficial. Additionally, exploring the mechanistic pathways linking astrocyte activation to amyloid and tau pathology will deepen our understanding of AD etiology and to what extent astrocyte activation is genetically regulated. Finally, newer tools allowing for more robust quantification of astrocyte activation *in vivo* using positron emission tomography tracers could serve as a complementary approach to the transcript signature leveraged here and increase statistical power in future studies [21].

### 4.5. Strengths and weaknesses

Our study has numerous strengths. We leveraged multiple well-characterized, deeply phenotyped cohort studies of aging to first determine the genetic architecture of astrocyte activation and then validate a PRS in predicting clinically relevant outcomes. Incorporating longitudinal measures of cognition and both amyloid and tau biomarker outcomes in our analyses allowed us to survey associations across the amyloid/tau/neurodegeneration framework. Despite its strengths, our study has notable weaknesses. Primarily, we were underpowered at the GWAS level due to the nature of building the astrocyte activation transcript signature from mRNA transcript sequencing from post-mortem brain tissue. Harmonization of brain transcriptomics across cohorts will enable higher-powered analyses in the future. Our study was also limited to individuals of Western European ancestry, limiting the generalizability of our findings to more diverse populations. We will be better equipped to investigate the utility of an astrocyte activation PRS in diverse populations as more data becomes available. In addition, we chose to employ a data-driven approach leveraging a previously published transcript signature of astrocyte activation [33]. However, a theory-driven approach could provide additional opportunities for discovery. Notably, key astrocyte genes known to be upregulated in reactive states were excluded from the transcript signature we used in our analyses. Potential candidates include: *GFAP*, *Serpina3n*, *VIM*, *AQP4*, and *Lcn2*, which are commonly upregulated in reactive astrocytes [34]. Future analyses incorporating such genes into the gene module will allow us to evaluate whether the inclusion of additional genes captures more of the polygenic architecture of astrocyte reactivity and improves the predictive ability of the PRS. Furthermore, the p-value cutoff used for PRS, though strongly correlated with the astrocyte activation transcript signature itself, was selected somewhat arbitrarily. This less-restrictive cutoff likely includes variants with smaller effects, which collectively may explain a large portion of variance in the phenotype. On the other hand, this may increase the risk of overfitting through the inclusion of more SNPs. Newer tools that enable fine-tuning of p-value cutoff selection for PRS will improve statistical power and predictive ability in future analyses. Furthermore, Since LD structure in the dataset used to build the PRS is likely playing a critical role in the relationship between the PRS and the astrocyte activation phenotype, assessing different R^2^ thresholds when using meta-analysis results leveraging multiple cohorts will be an important part of future work. Finally, none of the observed associations survived correction for multiple comparisons, potentially due to the GWAS’s power and sample size constraints. This will also be aided by the ever-increasing availability of brain transcriptomic measures and genetic data.

### 4.6. Conclusions

In summary, our study supports the potential role of an astrocyte activation PRS in predicting cognitive decline and AD biomarker burden. These findings emphasize the importance of astrocyte reactivity in AD progression and highlight the potential of genetic tools in early disease detection and personalized medicine. Further research and validation in well-powered datasets are needed to fully characterize the clinical utility of an astrocyte activation PRS in treating AD.

## Supplementary Material

Supplemental Figures

## Figures and Tables

**Table 1.** Participant Demographics

ROS/MAP	
Sample Size	598
Age at death (years)	81.1 +/− 6.97
Education (years)	16.53 +/− 3.5
Astrocyte Activation Z Score	0 +/− 0.61
Female, no. (%)	391 (65%)
Amyloid Positive at Autopsy, no. (%)	383 (64%)
Tau Positive at Autopsy, no. (%)	340 (57%)
AD diagnosis at last visit, no. (%)	252 (42%)
ADNI	
Sample Size	791
Age at baseline (years)	75.31 +/− 7.39
Education (years)	16.03 +/− 2.84
Total number of visits	6.32 +/− 2.93
Longitudinal follow-up (years)	4.89 +/− 3.51
Female, no. (%)	342 (43%)
Amyloid Positive at baseline, no. (%)	527 (67%)
Tau Positive at baseline, no. (%)	385 (49%)
AD diagnosis at baseline, no. (%)	196 (25%)

**Table 2.** PRS Main Effect Model Results

Outcome	β	p

Memory at baseline	−0.07	**0.03**
Executive function at baseline	−0.02	0.43
Language at baseline	−0.03	0.22
Longitudinal memory	−4.6E-3	0.43
Longitudinal executive function	−0.01	**0.03**
Longitudinal language	−2.3E-3	0.67
CSF Aβ1–42 at baseline	−3.4	0.07
CSF total tau at baseline	−0.29	0.87
CSF pTau at baseline	0.68	0.43

**Table 3.** PRS-Aβ1-42 Interaction Model Results

Outcome	β	p

Memory at baseline	3.7E-4	0.46
Executive function at baseline	2.8E-4	0.54
Language at baseline	3.7E-4	0.37
Longitudinal memory	-7.9E-6	0.93
Longitudinal executive function	1.2E-4	0.13
