# Supplementary material for: Astrocyte Reactivity Polygenic Risk Score May Predict Cognitive Decline in Alzheimer’s Disease
Source: Pac Symp Biocomput. Author manuscript; Available in PMC 2025 Jan 22. (PMC11752824; doi:10.1142/9789819807024_0035)
Supplement: Supplemental Figures [file NIHMS2038220-supplement-Supplemental_Figures.pdf]

Supplemental Material

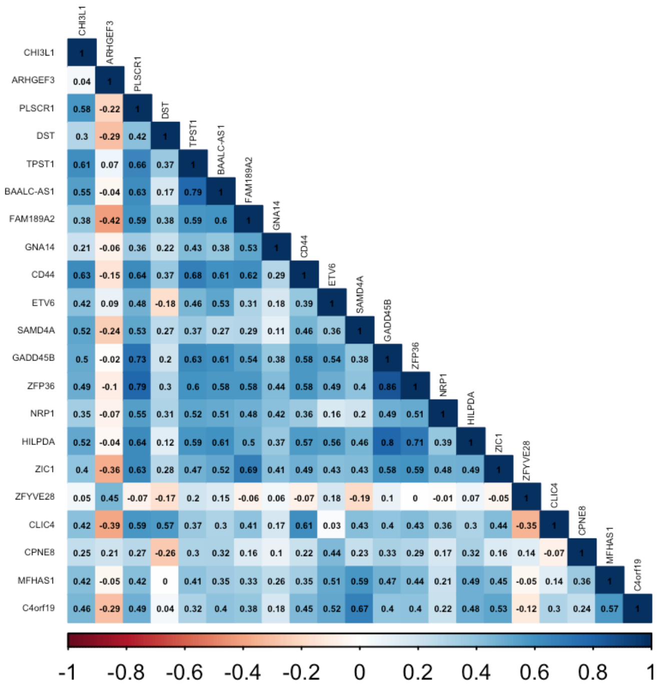

Supplemental Figure 1. Correlation of dorsolateral prefrontal cortex bulk expression of genes included in the astrocyte activation transcript signature.

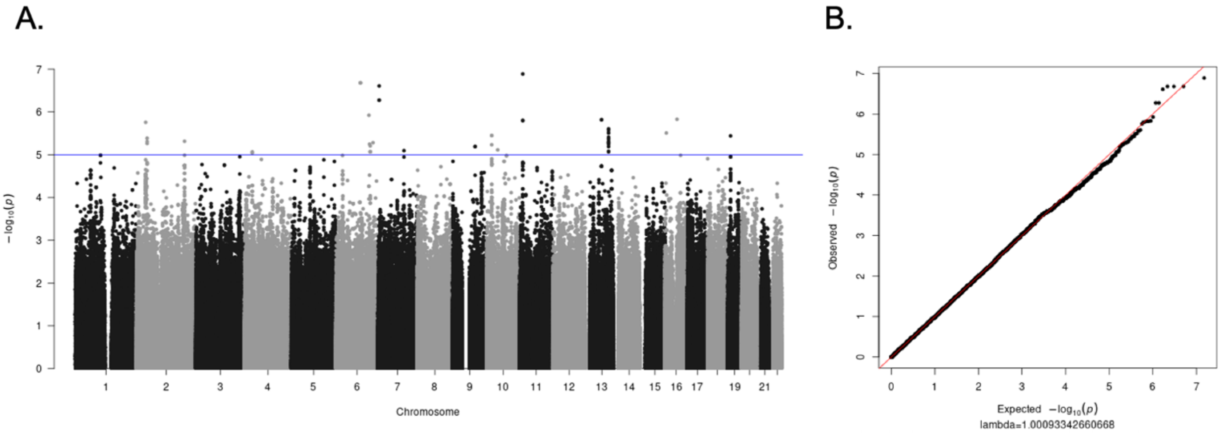

Supplemental Figure 2. A) Manhattan plot depicting variant associations with the astrocyte activation transcript signature in ROS/MAP. B) QQ plot of GWAS results.

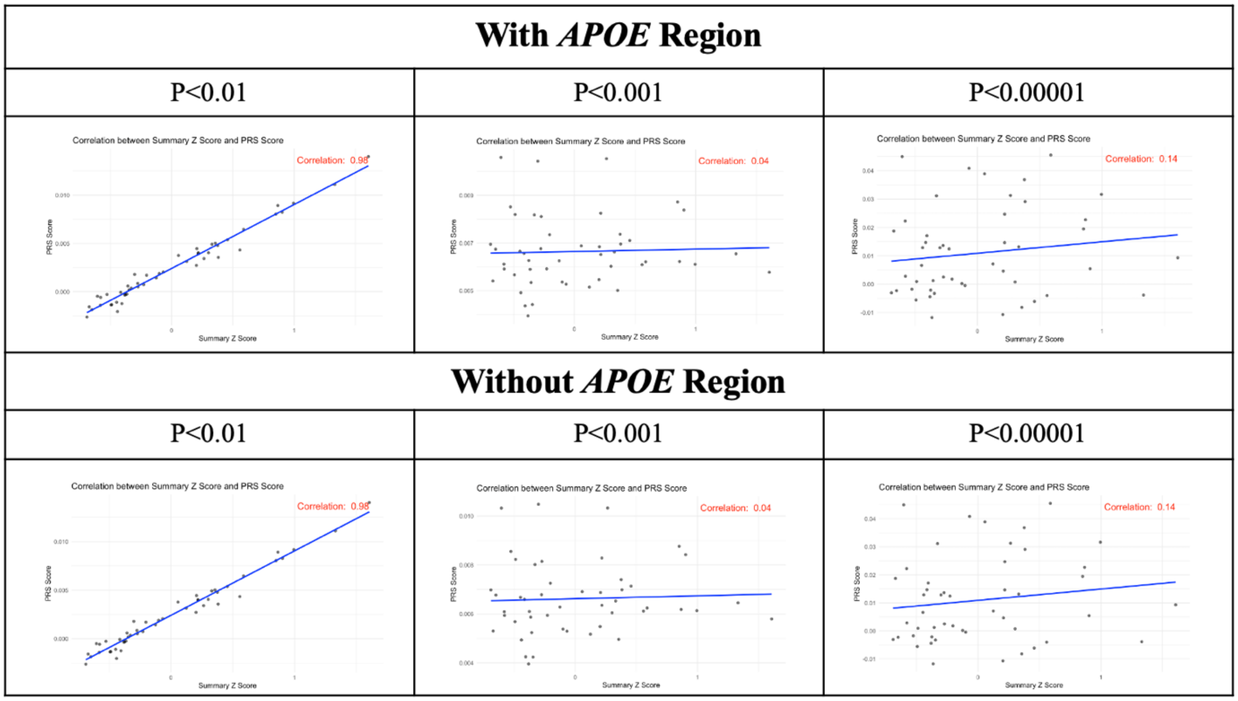

Supplemental Figure 3. Panels depicting the correlations between PRS calculated with and without the APOE region at various p-value cutoffs and the astrocyte activation transcript signature (Z-score).

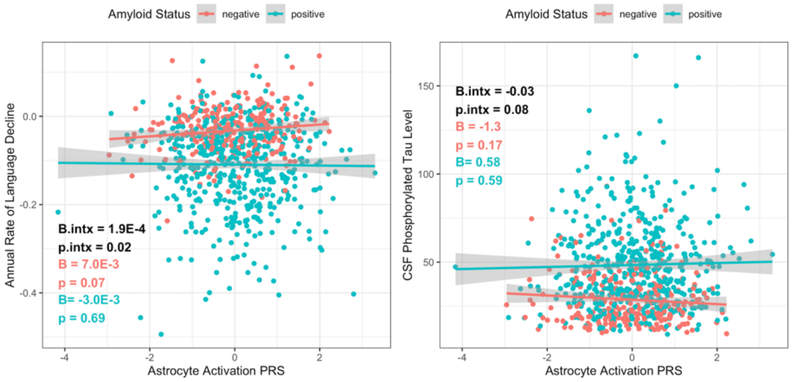

Supplemental Figure 4. Sensitivity analysis of PRS-Aβ42 interactions on annual rate of language decline and CSF phosphorylated tau. Interaction model statistical results are shown in black while amyloid-stratified main effect statistics are shown in colors corresponding to each stratification on the plot. Amyloid positivity was determined using Gaussian mixture modeling, with amyloid positivity defined as CSF β-amyloid(1–42) concentrations lower than 195 pg/mL; amyloid positive N = 520, amyloid negative N = 264).
